# A Case of Pancreatic Neuroendocrine Tumor with Liver Metastases Demonstrating the Possibility of Enhanced ACTH Production by the SACI Test

**DOI:** 10.1155/2024/5923680

**Published:** 2024-04-20

**Authors:** Hirozumi Mori, Masashi Tamura, Ryo Ogawa, Yuta Kimata, Sho Endo, Katsutoshi Sekine, Sayuri Kodama, Hiromi Hisazumi Watanabe, Kiyoshi Ookuma, Masahiro Jinzaki

**Affiliations:** ^1^Department of Radiology, Saitama City Hospital, Saitama, Japan; ^2^Department of Radiology, Keio University School of Medicine, Tokyo, Japan; ^3^Department of Medicine, Saitama City Hospital, Saitama, Japan

## Abstract

**Objective:**

ACTH-producing pancreatic NETs have a propensity to metastasize, and in patients with metastases, there is no established method yet to precisely determine if the excess ACTH is produced by the primary or the metastatic tumors. Localizing the source of production of ACTH in such cases is important for devising suitable treatment strategies and evaluating the benefit of local therapies from the viewpoint of control of Cushing's syndrome.

**Methods:**

We performed the selective arterial calcium injection (SACI) test combined with selective portal and hepatic venous sampling in a 32-year-old female patient with ectopic ACTH-producing pancreatic NET and liver metastases.

**Results:**

The blood level of ACTH after Ca loading was significantly elevated only in the vessels thought to be directly feeding the pancreatic tumor, and Ca loading from any artery did not significantly increase ACTH concentrations in the hepatic veins compared to the main trunk of the portal vein.

**Conclusions:**

The present case demonstrates that there might be an ACTH-producing p-NET that responds to Ca loading. Further in vitro studies are required to validate this possibility.

## 1. Introduction

ACTH is a hormone that is synthesized in the anterior pituitary lobe and regulates cortisol production in the adrenal gland. However, ectopic ACTH secretion, often resulting in Cushing's syndrome, by tumors arising outside the pituitary gland has also been reported [1]. Ectopic ACTH-producing tumors of the pancreas, in most cases pancreatic neuroendocrine tumors (pNETs), account for about 4%–16% of all ectopic ACTH-producing tumors [2].

Cushing's syndrome caused by ACTH-producing neuroendocrine tumors of the pancreas carries a poor prognosis because the hypercortisolism predisposes to infection, bone fractures, and electrolyte imbalances that worsen the patient's general condition [3], making surgical resection of the tumors in the early stages difficult [4]. Therefore, as a therapeutic goal, control of cortisol secretion is as important as the local control of tumor growth.

Metastases from neuroendocrine tumors are known to show altered characteristics, such as the expression of altered somatostatin receptor subtypes [5], which are targets for drug therapy [6], and production of hormones differing from those secreted by the primary tumors [7, 8]. In addition, positive immunostaining of the tumor cells for ACTH does not necessarily prove ACTH secretory capacity of the tumor cells [9]. Therefore, it is often difficult to determine if control of cortisol production can be achieved by resection of the primary tumor or resection of the metastatic tumors or if systemic therapies might be required. Determination of the source of hormone production prior to treatment would be useful for selecting the most appropriate treatment strategies, especially for patients in poor general condition.

The selective arterial calcium injection (SACI) test is commonly used to localize the source of hormone production in patients with functional neuroendocrine tumors and is often performed as a test to identify hormone-secreting tumors in cases where the tumor cannot be localized by CT or ultrasonography or in cases with mixed functional and nonfunctional neuroendocrine tumors such as MEN type I insulinoma or gastrinoma [10, 11]. However, to the best of our knowledge, there are no reports yet of the usefulness of SACI test to localize the source of ACTH secretion in patients of pancreatic NETs with distant metastases. Herein, we present a case of pancreatic NET with multiple liver metastases, in whom the SACI test was performed and primary pancreatic neuroendocrine tumor possibly increased ACTH secretion by calcium loading.

## 2. Case Presentation

A 32-year-old pregnant woman was admitted to our psychiatry department with worsening of her depression during the pregnancy. After admission, fetal death was confirmed and the patient's progress was monitored, but there was little improvement in her depressive state, and her mental instability continued to worsen. Physical examination revealed a full moon face as well as facial acnes, which were considered to be Cushing's sign. Blood tests revealed abnormal glucose tolerance, hypertension, and hypokalemia, and furthermore, the patient showed worsening of generalized malaise and developed difficulty in walking. Radiographs obtained after the fall showed compression fractures from the 9th to 12th thoracic vertebrae.

**Figure 1:**
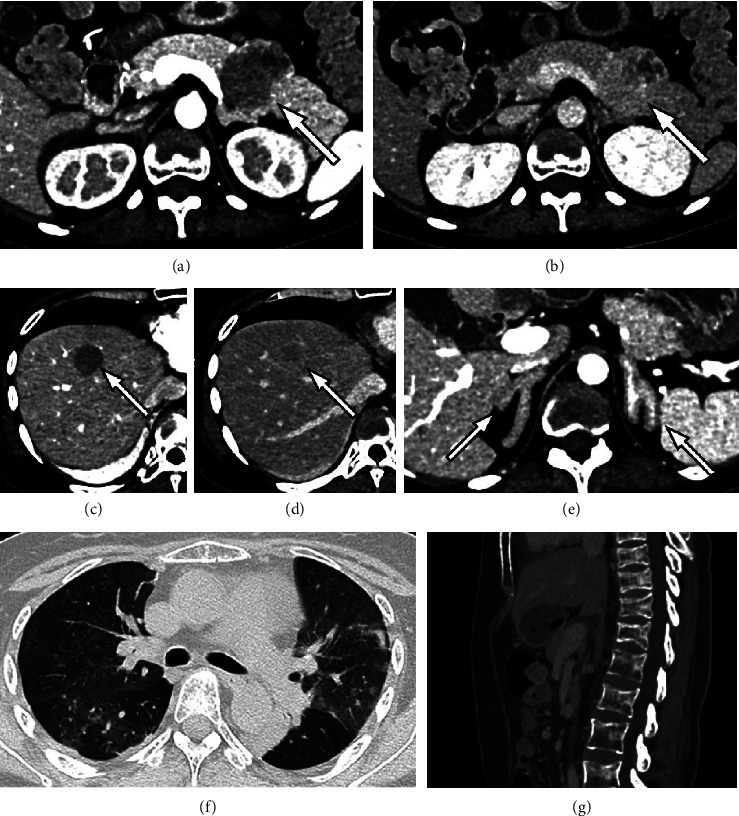
Findings of dynamic CT. (a-b) A tumor showing cystic degeneration was found in the pancreatic tail that showed gradually progressive staining from the arterial phase to the equilibrium phase. (c-d) Multiple liver metastases were also present in this case, one of which in the liver S8 showed the same contrast enhancement pattern as the pancreatic lesion. (e) Significant bilateral adrenal gland enlargement is seen. (f) Ground-glass appearance of both lungs with some infiltrative opacities, possibly suggestive of pneumocystis carinii pneumonia. (g) Compression fractures of the thoracolumbar spine due to osteoporosis.

While she was in the hospital, she fell and that evening, the patient's respiratory condition worsened and oxygen demand increased; therefore, we performed contrast-enhanced dynamic CT to rule out suspected pulmonary embolism, which revealed a progressively staining tumor in the pancreatic tail, multiple liver tumors showing similar contrast effects to those of the pancreatic tumor, marked adrenal enlargement, osteoporotic changes, and ground-glass appearance and infiltrative opacities in both lungs (Figure 1). Cushing's syndrome and associated infection with pneumocystis carinii pneumonia was suspected.

Blood samples showed elevated levels of ACTH (349 pg/mL) and cortisol (116 *μ*g/dL), consistent with ACTH overproduction, suggesting that the Cushing's syndrome in the patient was caused by ectopic ACTH production by the pancreatic neuroendocrine tumor. The depressive symptoms were considered as arising from psychosis secondary to hypercortisolemia.

**Figure 2:**
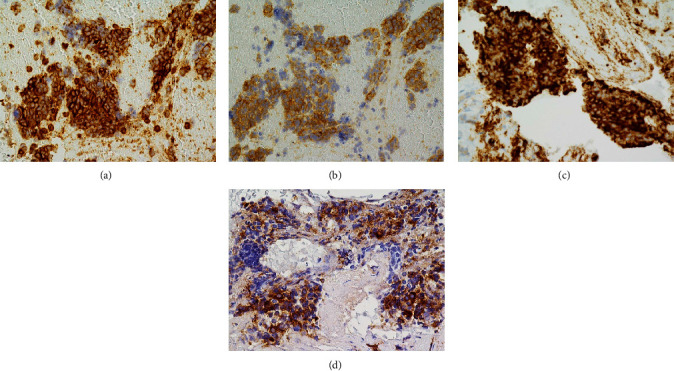
Immunohistochemical examination of pancreatic tumor. ACTH-secreting pNET showing positive immunostaining of the tumor cells for SSTR2 (a), SSTR5 (b), Chromogranin A (c), and ACTH (d).

On the basis of the histopathological findings obtained by EUS-FNA, we diagnosed the patient as having a neuroendocrine tumor (keratin AE1/AE3(+), keratin CAM5.2(±), CK7(−), chromogranin A(+), synaptophysin(+), CD56(+), CEA(−), and CD117(KIT) (±)). In addition, some tumor cells showed positive immunostaining for ACTH, strongly positive staining of the cell membrane for SSTR2, and slightly weaker staining for SSTR5, but positive staining of the cell membranes was observed in more than 50% of tumor cells (Figure 2). In the Volante's methods, both staining results of SSTR were assigned a score of 3 [12]. More than 2% but less than 20% of the cells were Ki-67-positive, and we diagnosed the tumor as G2.

**Figure 3:**
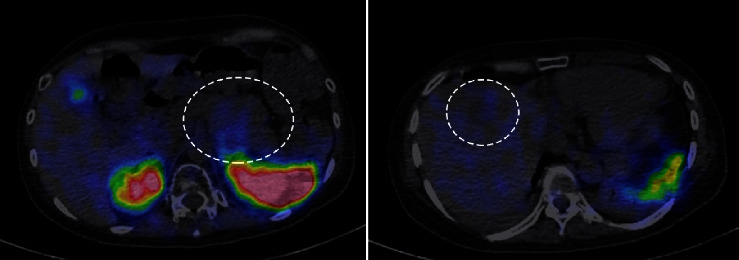
Octreoscan image. No significant accumulation was observed in the pancreatic tumor or liver metastases.

Based on the abovementioned findings, we diagnosed the patient as having a pancreatic ectopic ACTH-producing NET with liver metastasis. An Octreoscan (111In-pentetreotide SPECT) performed to investigate the usefulness of octreotide acetate and intranuclear therapy [13] revealed no significant accumulation in either the pancreatic lesion or the liver metastases (Figure 3) (scintigraphy with DOTATOC (68Ga-DODATOC PET), which is thought to be more sensitive for detecting metastatic NETs than Octreoscan [14] is not yet popularly used in Japan).

We then discussed the treatment plan based on the diagnosis of pancreatic neuroendocrine tumor with liver metastasis and ectopic ACTH production, as described above. The first treatment goal was to treat the potentially fatal pneumocystis pneumonia and improve the patient's general condition by improving the compromised immunity, electrolyte imbalances, muscle weakness, and osteoporosis associated with the high cortisol levels in the blood. We considered the following treatment options; oral therapy with steroid synthesis inhibitors for the hypercortisolemia, or with somatostatin analogs which exhibit antitumor effects [6], or local therapy, such as resection of the tumor or radiation therapy to abolish endocrine secretion from the tumor.

Because of the presence of metastases, it was necessary to localize the source of ACTH production to determine the usefulness of local treatment. Immunohistochemistry is considered as being insufficient to identify the localization of ACTH production [9] and evaluation by venous sampling is necessary. As a venous sampling technique, we decided to conduct blood sampling from the main trunk of the portal vein and the hepatic veins with selective arterial calcium injection to evaluate the production ACTH from the pancreatic tumors and liver metastases.

### 2.1. Portal Vein Sampling and Selective Arterial Calcium Injection Test

**Figure 4:**
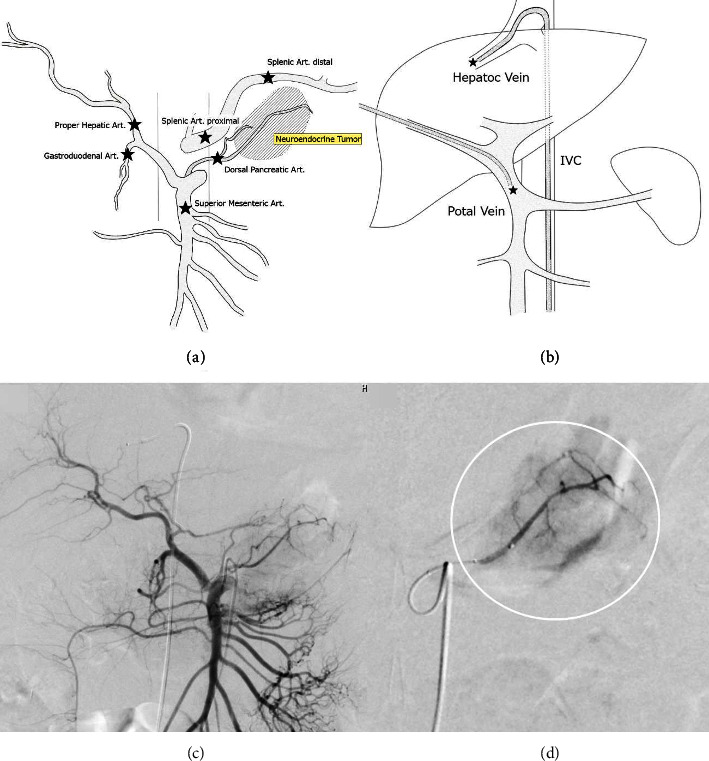
Schema for the SACI test and angiography. (a) Schema for the selective arterial calcium injection (SACI) test. Stars indicate the arteries tested in the current patient. (b) A catheter was inserted from the femoral vein into the middle hepatic vein and a catheter was inserted percutaneously and placed transhepatically into the main trunk of the portal vein, and the ACTH concentrations were measured. (c) SMA angiography. The replaced common hepatic artery (CHA) and dorsal pancreatic artery (DPA) are depicted. (d) Selective angiography from DPA showed that the pancreatic tumor was stained in a basket shape.

A 5Fr. catheter was inserted into the right femoral vein, and after drawing blood from the proximal and distal portion of the inferior vena cava before the calcium injection, it was placed in the middle hepatic vein (MHV), which was considered as the main drainage vein for the hepatic metastasis. A 4Fr. catheter was inserted percutaneously and was placed transhepatically into the main trunk of the portal vein (PV) (Figure 4(b)).

Superior mesenteric artery (SMA) angiography with a 4Fr. catheter indicated that the main feeding vessel for the tumor was the dorsal pancreatic artery (DPA) (Figures 4(c) and 4(d)). Using a microcatheter, the distal splenic artery (SPA dis), proximal splenic artery (SPA pro), proper hepatic artery (PHA), gastroduodenal artery (GDA), and dorsal pancreatic artery (DPA) were selected (Figure 4(a)) and after 0.025 mEq/kg (2.9 mL) of calcicol injection into each, blood samples were collected from the MHV and PV (preload, and 30, 60, 90, and 120 seconds postload). There was an interval of at least 10 minutes between each procedure, and the catheter was flushed with heparin-saline after each blood collection.

Finally, the puncture tract of the portal vein was embolized with coil.

### 2.2. Blood Sampling Results

**Figure 5:**
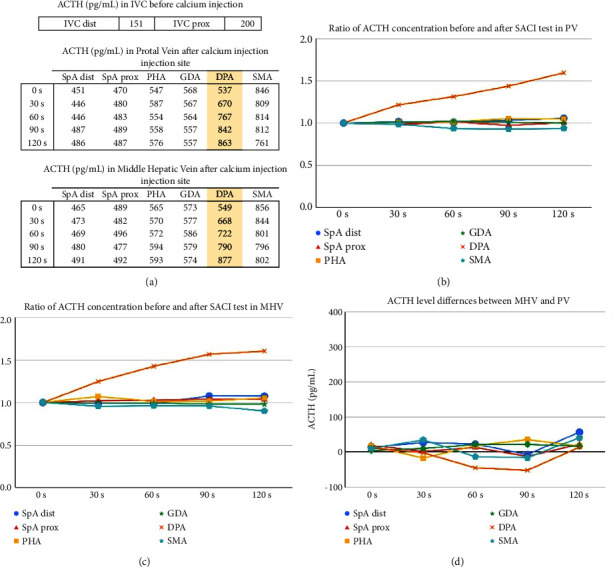
Results of the SACI test. (a) ACTH concentration in the inferior vena cava before the calcium injection: changes in the ACTH concentrations of the portal vein and middle hepatic vein after selective arterial calcium injection are shown. (b-c) The graph shows the ratio of ACTH concentration before and after the SACI test in PV and MHV. (d) Differences in the ACTH concentrations in the portal vein and middle hepatic vein after selective arterial calcium injection.

Comparison of the levels in the proximal and distal inferior vena cava prior to the calcium injection showed an increase in the ACTH concentrations, suggesting increased ACTH concentration in the portal or hepatic venous system (Figure 5(a)). The results of the selective arterial calcium injection showed that the injection into the DPA increased the ACTH concentrations in the PV and MHV (Figures 5(b) and 5(c)). Selective calcium injection into the other arteries did not result in any significant increase of the ACTH concentrations as compared with the preload values.

In addition, no significant ACTH concentration differences were observed in the MHV and PV after calcium loading of either artery (Figure 5(d)). These results did not provide any findings suggesting ACTH secretion from liver metastases.

Finally, these results indicated that ACTH secretion from pancreatic tumors could be induced by calcium injection.

### 2.3. Course of Treatment

In the present case, surgical resection of the pancreatic tumor was planned initially for the control of ACTH, but as the patient's general status was poor, lanreotide, a somatostatin analog with affinity for SSTR2/SSTR5 [15] was administered as prior therapy to local treatment with the aim of reducing ACTH secretion and shrinking the tumor, but no decrease in the levels of either ACTH or cortisol was observed, and follow-up CT scan showed a slight increase in size of both the primary tumor and liver metastases. Therefore, metyrapone and osilodrostat, which are inhibitors of steroid synthesis in the adrenal cortex, were administered to control the excess cortisol secretion. This treatment resulted in restoration of the cortisol blood levels to within the normal range (6.24–18 *μ*g/dL).

**Figure 6:**
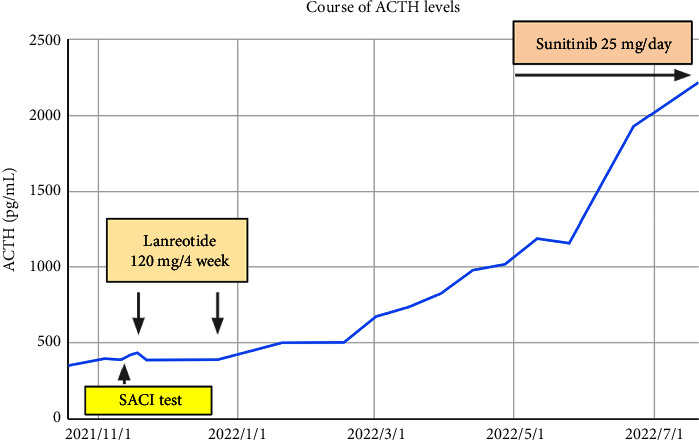
Changes in the ACTH level during the course of treatment. No decrease in the blood ACTH levels was observed after treatment with lanreotide or sunitinib.

In regard to the ACTH levels, ACTH production was not suppressed even after administration of sunitinib, which is a kinase inhibitor, and in fact, it shows a rapid increase following this treatment (Figure 6). As the cortisol levels decreased, the general condition of the patient transiently improved to the point where she was able to live independently without the need for assistance from others. Therefore, the timing of resection of the primary pancreatic lesion was explored, but the liver metastases increased in number and grew in size at a rapid rate, and the patient died 14 months after diagnosis, primarily due to liver failure.

## 3. Discussion

### 3.1. Portal Vein Sampling and Selective Arterial Calcium Injection Test in Patients with Ectopic ACTH-Producing pNETs

Since there are some reports of neuroendocrine tumors which changed their hormone-secreting capacity after the development of metastases [8] (for example, there is a report of a gastrin-producing tumor of the pancreatic head that presented with ectopic ACTH syndrome after the development of liver metastasis) [7], it would be useful to evaluate the hormone-secreting capacity of metastases from the viewpoint of control of cortisol secretion.

In this case, liver metastasis was confirmed on dynamic CT, and it was important for us to determine the source of ectopic ACTH production and whether resection of the primary pancreatic tumor would be meaningful for cortisol control. The portal vein sampling (PVS) and SACI test were performed as described above to localize the origin of ACTH production.

PVS has been reported as a selective venous sampling method for pancreatic neuroendocrine tumors (NETs) [16–18]. Roche et al. [18] successfully identified tumors in 36 out of 38 cases undergoing surgery for insulinomas or gastrinomas. They reported no case of incorrect tumor localization by PVS. In addition, they noted that 27% of insulinomas and 43% of gastrinomas localized by PVS were not detectable during gross examination during surgery but microscopic tumors were identified. As the less invasive SACI test for insulinomas and gastrinomas has been subsequently established [19, 20], the use of PVS is now less common.

The significance of selective venous blood sampling in the localization of ectopic ACTH-producing tumors has been reported at various sites [5, 20–24]. Doppman et al. [24] reported that wide-ranging venous sampling of systemic and portal veins was negative in the absence of tumor identification on other imaging, but in two cases of ACTH-producing pheochromocytoma noted on CT, the localization was successfully identified by selective venous sampling. Regarding ACTH-producing pancreatic NETs, Byun et al. [23] reported a case of ACTH-producing NET in the pancreatic tail in which elevated ACTH concentration was confirmed by PVS and resection of the tumor in the tail resulted in symptomatic remission. Kondo et al. [5] performed hepatic venous blood sampling for pancreatic NETs with liver metastases and evaluated ACTH production.

PVS in this case was adopted as selective venous sampling that could assess the secretion of ACTH from the primary pancreatic tumor. In addition to PVS, we also simultaneously performed hepatic venous sampling to evaluate the secretion of ACTH from the metastatic liver tumors. It was considered that ACTH secretion from the primary pancreatic tumor could be evaluated by comparing the ACTH concentrations in the PV and IVC and that ACTH secretion from the metastatic liver tumors could be evaluated by comparing the concentrations in the PV and MHV even if calcium injection did not induce ACTH production from the pancreatic tumor. As a result, it was suggested that the primary pancreatic tumor was more likely to be producing ACTH in this case and that there might exist pancreatic NETs where ACTH production is stimulated by Ca loading.

Conventionally, more than 2-fold increase after calcium stimulation in the serum insulin concentration over its baseline value is considered to be positive for pancreatic insulinoma [25] although more than 1.2-fold increase after calcium stimulation in the serum gastrin concentration over its baseline value is considered to be positive for pancreatic gastrinoma [26–28]. In this case, as shown in Figure 5, only Ca infusion into DPA resulted in an increase in ACTH levels of more than 1.6 fold, whereas Ca infusion into other arteries showed little change in ACTH levels. After multidisciplinary discussions, our team judged that the increase in ACTH from the tumor was enhanced after Ca infusion into the DPA in this case.

In the present study, ACTH was found to increase in the portal vein and hepatic vein after Ca loading by SACI test. This suggests a mechanism by which tumor cells increase ACTH secretion upon Ca loading. However, at this point, there is no literature that has elucidated the response of ACTH-producing pancreatic NETs to Ca loading or the existence of Ca receptors, so what can be said about the mechanism is limited and can only be speculated. Previous literature has reported the presence of Ca receptors in insulinomas [29] and gastrinomas [30], which are endocrine tumors of the pancreas, and it has been reported that Ca loading stimulated insulin secretion in insulinomas by ex vivo cell culture [31]. It is hoped that similar knowledge will be accumulated for ACTH-producing pancreatic NETs in the future to establish a theoretical basis.

In addition, surgical resection which confirms a decrease in ACTH concentration could not be performed, and the possibility of a false negative result from liver metastases could not be excluded, but the comparison of ACTH concentrations in the hepatic vein and portal vein did not provide any findings suggesting ACTH secretion from liver metastases. The present study suggests that the SACI test could promote ACTH secretion from pancreatic tumors. However, as mentioned above, theoretical support for increased ACTH secretion in ACTH-producing pancreatic neuroendocrine tumors by calcium loading is lacking at present, so it is desirable to establish theoretical support in the future through in vitro validation.

### 3.2. Considerations to Explain the Dissociation between STR2/SSR5 Positivity and Octreoscan Results on Immunostaining and Reactivity to Therapeutic Agents

In the present case, immunohistochemistry revealed strongly positive staining of the pancreatic tumor tissue for SSTR2 and SSTR5, which are subtypes of somatostatin receptors, but no accumulation was observed on Octreoscan. This might suggest that while the antibodies bound to SSTR, octreotide did not bind stably or effectively to this receptor. In fact, the binding sites of antibodies and octreotide to the SSTR2 structure are known to be different [32–34]. Since existence of a correlation has been reported between the efficacy of drugs in intranuclear therapy for NETs and the accumulation on Octreoscan [13], and since somatostatin analogs share a common binding site to octreotide and SSTR2 [34], improved binding of octreotide to SSTR may improve the prognosis of patients with neuroendocrine tumors.

In conclusion, the present case demonstrates that there might be an ACTH-producing p-NET that responds to Ca loading. Further in vitro studies are required to validate this possibility.
